# Supplementary material for: Urinary signatures are associated with calorie restriction-mediated weight loss in obese Diversity Outbred mice
Source: PLoS One. 2025 Dec 9;20(12):e0329422. doi: 10.1371/journal.pone.0329422 (PMC12688116; doi:10.1371/journal.pone.0329422)
Supplement: S1 Table — Two-Way ANOVA followed by FDR correction using MetaboAnalyst was performed to determine sex-specific urinary metabolites. (DOCX) [file pone.0329422.s001.docx]

|  | Responder vs Nonresponder (raw p-value) | Responder vs Nonresponder (adj. p-value) | Sex  (raw p-value) | Sex  (adj. p-value) | Interaction (raw p-value) | Interaction (adj. p-value) |
| --- | --- | --- | --- | --- | --- | --- |
| Histamine | 0.024 | 0.074 | 1.6X10^-5^ | 1.9X10^-4^ | 0.012 | 0.44 |
| Glutamic acid | 8.6X10^-4^ | 0.008 | 0.003 | 0.0079 | 0.036 | 0.49 |
| Putrescine | 0.013 | 0.051 | 2.1X10^-5^ | 1.9X10^-4^ | 0.097 | 0.49 |
| Leucine | 0.19 | 0.33 | 1.4X10^-4^ | 5.9X10^-4^ | 0.13 | 0.49 |
| Methionine | 0.21 | 0.33 | 6.4X10^-5^ | 3.4X10^-4^ | 0.15 | 0.49 |
| Glutamine | 0.68 | 0.79 | 1.0X10^-4^ | 4.6X10^-4^ | 0.1 | 0.49 |
| Asymmetric dimethylarginine | 0.9 | 0.94 | 5.9X10^-6^ | 1.0X10^-4^ | 0.07 | 0.49 |
| Histidine | 0.91 | 0.94 | 2.6X10^-5^ | 1.9X10^-4^ | 0.15 | 0.49 |
| Dopamine | 1.5X10^-5^ | 5.8X10^-4^ | 0.97 | 0.97 | 0.24 | 0.53 |
| Spermine | 2.0X10^-4^ | 0.0028 | 0.005 | 0.012 | 0.22 | 0.53 |
| Asparagine | 0.01 | 0.046 | 0.066 | 0.11 | 0.22 | 0.53 |
| cis-4-Hydroxyproline | 2.3X10^-4^ | 0.0028 | 0.096 | 0.14 | 0.34 | 0.68 |
| Tyrosine | 0.35 | 0.46 | 0.011 | 0.025 | 0.46 | 0.74 |
| trans-4-Hydroxyproline | 0.007 | 0.043 | 9.0X10^-4^ | 0.0025 | 0.654 | 0.84 |
| Glycine | 0.071 | 0.18 | 1.0X10^-6^ | 3.8X10^-5^ | 0.71 | 0.84 |
| Proline | 0.1 | 0.24 | 0.02 | 0.043 | 0.75 | 0.84 |
| Symmetric dimethylarginine | 0.12 | 0.28 | 3.7X10^-5^ | 2.3X10^-4^ | 0.64 | 0.84 |
| Serotonin | 0.33 | 0.46 | 2.3X10^-4^ | 8.6x10^-4^ | 0.69 | 0.84 |
| Spermidine | 0.9 | 0.94 | 6.3X10^-4^ | 0.0021 | 0.79 | 0.84 |
| Dihydroxyphenylalanine | 0.002 | 0.014 | 9.0X10^-4^ | 0.0025 | 0.87 | 0.89 |
| N-Acetylornithine | 0.01 | 0.046 | 0.311 | 0.39 | 0.92 | 0.92 |
